# Primary Cutaneous B-Cell Lymphomas in Patients With Impaired Immunity

**DOI:** 10.3389/fonc.2020.01296

**Published:** 2020-09-11

**Authors:** Irene Russo, Laura Fagotto, Alvise Sernicola, Mauro Alaibac

**Affiliations:** Unit of Dermatology, University of Padua, Padua, Italy

**Keywords:** cutaneous B-cell lymphoma, immunosuppression, posttransplant lymphoproliferative disorder, EBV, HIV

Primary cutaneous B-cell lymphomas (CBCL) are a group of non-Hodgkin lymphomas (NHL) defined by a clonal B-cell proliferation involving the skin and no extracutaneous districts and constitute 20–25% of primary cutaneous lymphomas (1). The “2018 update of the WHO-EORTC classification” recognized three subtypes: primary cutaneous marginal zone lymphoma (PCMZL), primary cutaneous follicle center cell lymphoma (PCFCCL), and primary cutaneous diffuse large B-cell lymphoma, leg type (PCDLBCL, LT) (2). A diagnosis of PCDLBCL, NOS, covers rare cases that are not classifiable as either PCDLBCL, LT or PCFCL.

PCMZL is an indolent form of lymphoma that accounts for up to 30–40% of primary cutaneous lymphomas. PCMZL tends to relapse in the skin, but dissemination to extracutaneous sites is rare and the prognosis is excellent, with an estimated five year survival rate of 99% (3). PCFCCL is an indolent lymphoma that comprehends ~40–50% of primary CBCLs. PCFCCLs can recur in up to 30% of patients, but dissemination to extracutaneous sites is rare and survival is over 95% at 5 years (2). PCMZL and PCFCCL are characterized by an indolent behavior; but PCDLBCL, LT is instead characterized by an intermediate to aggressive clinical behavior. PCDBCL, LT occurs most commonly with skin lesions on the lower legs, accounts for under 20% of primary CBCL and affects elderly, predominantly female patients. Compared to the other subtypes of CBCL, this lymphoma disseminates to extracutaneous sites more often and has a more unfavorable prognosis, with a 5 year survival rate of around 55%.

EBV+ mucocutaneous ulcer (EBVMCU) is a provisional type of indolent B-cell lymphoma that occurs in cases of immunosuppression due to treatment or age (4). CBCL are rare entities in both immunocompetent and immunosuppressed patients. However, these two groups of patients show different subtypes, with different clinical behavior and prognosis. In immunocompetent patients primary CBCL usually have an indolent behavior is typical of PCMZL and PCFCCL. They also have a favorable outcome, with survival rates above 95% after 5 years. Aggressive subtypes are very rare in this group. Even if immunosuppression is a known risk factor in lymphoproliferative disorders, it is difficult to compare the different causes of immunosuppression. However, in all types of immunosuppression, aggressive subtypes of CBCL are observed more frequently than in immunocompetent patients, while indolent subtypes are virtually non-existent (5, 6).

We focused on CBCL in two main contexts of immunosuppression, iatrogenic and non-iatrogenic, to highlight the role of immune surveillance in determining the behavior of these disorders. Different subtypes of CBCL are associated to different prognosis, which could be due to how CBCL interact with the immune system and whether it is functioning or impaired.

Although epidemiological data on primary cutaneous lymphomas in immunocompromised patients is scarce, there are case series and isolated reports on HIV and EBV infections in some congenital immunodeficiency disorders and in iatrogenic immunosuppression. Non-iatrogenic immunosuppression may occur when infectious agents impair host immune response. The immunological events induced by these agents, notably EBV, HIV and *Borrelia* (6–8), are responsible for different types of immune dysregulation. Even though it is difficult to compare these different types of immune dysregulation, this overall response provides insight into the role of an efficient immune system in the surveillance for malignant cells and oncogenic pathogens.

## Non-Iatrogenic Immunosuppression

### CBCL and EBV Infection

EBV is a ubiquitous virus, capable of establishing a persistent infection that remains subclinical in the majority of individuals. This viral latency involves a complex homeostasis in the host, created by the pathogen's mechanisms of immune and host surveillance evasion (7). EBV selectively colonizes host B-cells and is able to persist in EBV+ memory B-cells, which become the viral reservoir. In the initial form of latency infected B-cells display a growth-transformation phenotype. They also express a wide array of EBV latent proteins, leading to immune recognition by cytotoxic T-cells. Viral latent membrane proteins provide the infected B-cells with anti-apoptotic and proliferative signals, causing cell immortalization and increasing the risk of developing malignant B-cell proliferation (9). In immunocompetent carriers, the effective immune clearance of EBV-infected cells leads to forms of deeper latency that also evade host surveillance, meaning EBV is a silent infection. Ineffective viral clearance is common when immunity is suppressed due to concurrent infection, iatrogenic causes, and mutations responsible for the syndrome.

Specific types of primary immunodeficiency suggest potential strategies to treat EBV-induced conditions, as they provide unique models for understanding the immune mechanisms required to control EBV infection. Abnormal susceptibility to EBV is associated to X-linked lymphoproliferative disease (10), Wiskott-Aldrich syndrome (11) and different defects responsible for combined immunodeficiency. Absence of normal T-cell function in severe combined immunodeficiency exposes patients to EBV-induced B-cell lymphoproliferation at an early age (12). On the other hand, common variable immunodeficiency (CVID) causes B-cell defects that prevent plasma cell differentiation and antibody secretion together with variable defects in T-cells and dendritic cells.

Though prominent EBV infections are not typical of CVID, increased risk of malignancy due to B-cell lymphoma is occasionally associated with EBV (13). EBV-associated lymphoproliferations rarely present to the skin. Cases of CBCL have been reported in the setting of iatrogenic immunosuppression and DLBCL, NOS, is the most common subtype. These cases are associated to prolonged administration of drugs, leading to years of immune dysregulation before the onset of CBCL (14).

In subjects with no known cause of immune suppression, an association with EBV has been demonstrated in rare types of CBCL, with positive *in situ* hybridization of EBV. Primary cutaneous EBV+ DLBCL is an aggressive B-cell lymphoma that has been reported in immunocompetent adults over 50 years old, and is associated with a poor prognosis. The expression of latent membrane proteins is associated to clonal B-cell proliferation, suggesting that these proteins have a role in B-cell immortalization (9). HHV-8, a gamma herpesvirus related to EBV, is capable of infecting B-cells and plasmablasts through virally secreted proteins which can stimulate proliferation of B-cells and plasma cells. In certain cases, HHV-8 induces a B-cell plasmablastic lymphoma, a rare subtype of DLBCL (15, 16). Cutaneous lymphomagenesis is related to HHV-8 infection and occurs in a setting of immunodeficiency in this variant, as well as in specific presentations of primary effusion lymphoma in the skin (17, 18).

### CBCL and HIV Infection

Lymphomas associated with HIV infection usually share advanced-stage presentation and an aggressive clinical course. Prior to the introduction of combination antiretroviral therapy (cART) the risk of NHL was over 100 times higher in AIDS patients. Following the introduction of cART, the relative risk is still around 10–20. The most aggressive subtypes, DLBCL and BL, remain common and are associated to a significantly reduced survival rate when compared to HIV-negative patients with the same diagnosis (6). Primary cutaneous lymphomas represent 10% of extranodal NHLs and have a B-cell origin in 15% of HIV patients. It has been reported that cases of isolated skin disease with no evidence of other localizations respond to cART alone, highlighting the role of immune reconstitution in the control of CBCL (19).

### CBCL and Borrelia

Persistent infection with *Borrelia burgdorferi (Bb)* and protozoal species of *Leishmania* may be the consequence of pathogen-induced immunosuppression (20, 21). The mechanisms of immunotolerance have been studied in murine models of *Bb* infection and include inhibition of T-cell activation and of effective antibody mediated response (8, 22). *Bb* infection has been associated with PCMZL in some European cases (23). Lymphomagenesis in the skin could be triggered by pathogen-induced immunosuppression or alternatively, by chronic inflammatory stimulation and persistent lymphocyte activation due to an inability to eliminate the pathogen (24).

In the late stage of Lyme borreliosis, lymphocyte infiltration in the dermis creates a “lymphocytoma” or cutaneous B-cell pseudolymphoma (24). As the histology of B-cell pseudolymphoma and PCMZL are similar (4, 25), some authors suggest that there is a progression from lymphocitoma and pseudolymphoma to clonal proliferation that conducts to PCMZL (24). The monoclonality of B-cells is a useful way to differentiate between pseudolymphoma and lymphoma (24). The association between *Bb* and PCMZL is supported by the remission of the disease after antibiotic therapy is used to treat the *Bb* infection. Despite the fact that many patients do not respond to this treatment, in some cases antibiotic therapy has led to the remission of *Bb* positive PCMZL (24, 25). Taking into account the indolent behavior of PCMZL, attempts to treat *Bb*-positive PCMZL with antibiotics should be considered as a first line therapy (25). The indolent behavior of CBCL, as observed in relation to *Bb* infection, supports the hypothesis that chronic antigenic stimulation is determined by the bacteria, which is in this case, the main mechanism causing lymphomagenesis. In chronic leishmaniosis, variable parasite-host interactions are responsible for a spectrum of cutaneous syndromes that may induce B-cell proliferation. However, bacterial association with CBCL has not as yet been clearly defined (26).

## Iatrogenic Immunosuppression

### CBCL and Transplant Patients

Iatrogenic immunosuppression is usually observed in transplant patients and is related to anti-rejection therapy. The risk of developing any kind of NHL increases over six-fold following organ transplantation and varies greatly according to subtypes. This increased risk of post-transplant lymphoproliferative disorders (PTLD) involves a specific spectrum of histologic subtypes and of associated risk factors. Aggressive subtypes of PTLD show strong elevations of risk, while only few cases of indolent lymphomas are reported in transplant recipients with no significant elevation in risk (27).

Extranodal disease is a common manifestation in these patients and the skin is a major localization (28). Seckin et al. (5) collected a multicenter European retrospective of primary cutaneous PTLD, of which 31.4% were primary CBCL. Of these primary CBCL, 90.9% were associated with EBV. The remaining 9.1% DLBCL, NOS were EBV-negative. In terms of patients, 27.3% from this series had a fatal outcome due to PTLD, which is exceptional considering that the prognosis of CBCL is more favorable than that of systemic forms. Risk factors associated to cutaneous B-cell PTLD are anti-rejection therapy and EBV infection. Different drug regimens influence the risk of PTLD.

Heart transplant recipients that require a high dose of immunosuppressive therapy were overrepresented in published cases, accounting for 36.4% of patients with B-cell PTLD (5). The association between EBV and B-cell PTLD is well established, with an estimated 50–70% rate of EBV-positivity discovered with FISH or PCR in tumor specimens (28). Chronic immune suppression impairs T-cell surveillance of EBV-infected B-cells and leads to the uncontrolled proliferation of EBV-transformed B-cells. This process also occurs in EBVMCU associated to iatrogenic immunosuppression. The reduction of therapy may be sufficient to achieve remission of this cutaneous EBV-related disorder (29). Several cases of aggressive subtypes of cutaneous B-cell PTLD have been reported, while indolent subtypes have not been observed in transplant recipients (5). The absence of the indolent forms of CBCL (PCMZL and PCFCCL) in this group of patients indicates that the development of these subtypes is probably hampered by immunosuppressive regimen. An analogous disproportion between aggressive and indolent forms of primary cutaneous lymphoma in immunocompromised patients, has been previously discussed in PTLD of T-cell origin (30).

## Conclusions

In conclusion, these observations in immunosuppressed patients highlight the difference between indolent and aggressive forms of CBCL. Indolent subtypes are generally not observed in immunocompromised subjects. This finding, together with the clinical behavior of indolent forms in immunocompetent subjects, suggests that these lymphoproliferative processes are induced by mechanisms closely related to chronic inflammation. On the other hand, aggressive forms of CBCL are generally observed in the context of immunosuppression, where they are favored by altered immune surveillance and oncogenic viral stimuli, and should be regarded as true lymphomas.

## Author Contributions

All authors listed have made a substantial, direct and intellectual contribution to the work, and approved it for publication.

## Conflict of Interest

The authors declare that the research was conducted in the absence of any commercial or financial relationships that could be construed as a potential conflict of interest. The reviewer EB declared a past co-authorship with one of the authors MA to the handling editor.
